# Immune alterations and overexpression of CTCF in endometrial carcinoma: insights from molecular subtyping

**DOI:** 10.1186/s12935-024-03576-y

**Published:** 2024-12-02

**Authors:** Caiping Wei, Guowei Chen, Kun Chen, Shuang Fang, Hongying He

**Affiliations:** 1 Department of Gynecology, Liuzhou Municipal Liutie Central Hospital, Liuzhou, Guangxi China; 2 Department of Gynecology, Liuzhou Worker’s Hospital, Liuzhou, Guangxi China; 3 Key Laboratory of Medical Molecular Diagnostics of Liuzhou, Key Laboratory for Nucleic Acid Molecular Diagnosis and Application of Guangxi Health and Wellness Commission, Liuzhou, Guangxi China; 4 Department of Gynecology, GuangZhou Women and Children’s Medical Center Liuzhou Hospital, Liuzhou, Guangxi China

**Keywords:** Endometrial cancer, Molecular subtyping, DEGs, CTCF

## Abstract

**Background:**

Endometrial cancer (EC) is a prevalent epithelial malignancy originating in the female endometrium, and its global incidence has been on the rise over the past decade. Despite significant scientific progress has been achieved recently, the genetic factors underlying EC pathogenesis remain poorly understood, warranting further investigation.

**Methods:**

We employed transcriptomic datasets from the Genomic Data Commons database to extract variable and clinical data. Quantile normalization and log2 transformations were applied to obtain a gene expression matrix for the sample cohort. Various assays, such as quantitative real-time polymerase chain reaction (qRT-PCR), western blotting, immunohistochemistry (IHC), wound healing assay, transwell assay, and TUNEL assay, were employed in the study to examine the involvement of CTCF in EC cell biology. Additionally, in vivo experiments were conducted using a subcutaneous transplantation tumor model in athymic nude mice. The potential mechanism of action of CTCF was also illustrated by identifying differentially expressed genes (DEGs) and functions after interfering with CTCF gene expression through the GSPAdb online database.

**Results:**

After categorizing 543 samples into cohorts with high and low ImmuneScores, we discovered 1025 genes that were differentially expressed, including 745 genes that were up-regulated and 280 genes that were down-regulated in the high scores group compared to the low scores group. Tumor mutation burden (TMB) analysis highlighted 11 genes with the highest mutation frequency. Furthermore, 16 immune checkpoints and 50 immune regulatory factors exhibited differential expression. Among these, CTCF was up-regulated in EC. We found that CTCF knockdown could diminish EC's invasive ability and metastatic potential while enhancing apoptosis. In vivo experiments corroborated that CTCF knockdown could reduce tumor growth. The GSPAdb online database identified differential expression pathways mainly enriched in cellular metabolism as well as some intracellular classical signaling pathways after interfering with CTCF gene expression. In addition, we identified potential downstream regulators of CTCF through protein interaction networks.

**Conclusion:**

This study unveiled comprehensive molecular characteristics and DEGs in EC, emphasizing the up-regulation of CTCF in EC. Our findings collectively suggest that CTCF represents a promising therapeutic target, and our gene molecular typing model offers a novel approach for prognostic evaluation in EC.

**Supplementary Information:**

The online version contains supplementary material available at 10.1186/s12935-024-03576-y.

## Highlights

1. 1025 differentially expressed genes, including CTCF, were identified in endometrial cancer (EC).
2. CTCF knockdown significantly mitigated the growth, invasiveness, and metastatic potential of EC.
3. CTCF could be a potential therapeutic target and prognostic tool for EC patients.

**Supplementary Information:**

The online version contains supplementary material available at 10.1186/s12935-024-03576-y.

## Introduction

Endometrial cancer (EC) is one of the three major gynecological malignancies, with 417,000 new cases diagnosed in 2020 worldwide [1, 2]. It is now the most prevalent gynecological malignant tumor in Europe and the United States, with 65,620 new cases and 12,590 subsequent deaths occurred in 2020 in the United States [3]. EC primarily occurs in postmenopausal women, and the average age at diagnosis is 63 years [4]. Diagnosis of EC relies on endometrial biopsy, section diagnosis, or hysteroscopy, with surgical treatment mainly determining the pathological stage and operation procedure [5]. Timely diagnosis and treatment in the early stages usually lead to a better prognosis, while patients diagnosed at later stages or with higher risk may require radiotherapy or chemotherapy [6]. Advances in clinical research and the standardization of diagnosis and treatment have significantly improved the prognosis of EC in recent years, with an overall survival rate up to 80% within five years [7].

Typically, the first subtype classification of EC is based on clinical and hormonal characteristics, distinguishing between Type I and Type II EC [8]. The etiology of EC is primarily estrogen-dependent, mainly presenting as Type I EC, which is associated with risk factors such as hypertension, diabetes, obesity, and delayed menopause [9]. These factors increase the risk of developing Type I EC, but Type I EC is relatively mild and generally has a better prognosis than Type II EC [10]. In contrast, Type II EC is not estrogen-dependent and is caused by genetic mutations [11]. It is characterized by more aggressive histologies, such as serous carcinoma and clear cell carcinoma, typically affecting older individuals and having a poorer prognosis [12]. Additionally, patients with Lynch syndrome, a hereditary condition, are at an increased risk of developing EC, along with other cancers [13].

The introduction of The Cancer Genome Atlas (TCGA) molecular typing has shifted EC classification from pure pathological categorization to a method combining immune characteristics and molecular changes, enhancing the precision and individualization of clinical treatment [14]. This advancement has huge significance in defining grade 3 endometrial adenocarcinoma and serous carcinoma. However, due to tumor heterogeneity, the evaluation of multiple molecular changes in the same tumor lacks a unified standard [15]. Further investigation should prioritize examining the correlation between alterations in EC molecular levels, prognosis, potential molecular therapeutic targets, and the development of targeted therapies with improved response rates for precise and personalized clinical intervention. This can be achieved through the utilization of multi-center studies and extensive prospective data involving large samples [16, 17].

Herein, we retrospectively analyzed 543 EC cases, systematically examining factors influencing patient prognosis through follow-up assessments. Our findings have the potential to reveal new candidate genes associated with EC treatment and explore potential mechanisms of action.

## Materials and methods

### Data sources

In the present study, 543 EC samples were analyzed, which were downloaded from TCGA databases.

### Immune scoring

First, RNA-seq expression data for EC samples were collected and collated, and it was ensured that the data were normalized. Then, using the ESTIMATE R package [18], the ImmuneScore, StromalScore, and tumor purity were calculated for each sample by the ESTIMATEScore function. The immunity score reflects the level of immune cell infiltration in the tumor microenvironment by analyzing the expression patterns of immune-related genes in the samples. After the calculation, the samples were divided into high-immunity and low-immunity groups based on the median of the immunity score, which was used for subsequent difference analysis and clinical relevance assessment to explore the relationship between the level of immune infiltration and disease characteristics or prognosis.

### Differential expression analysis

The R package limma was used to conduct a differential expression analysis on the mRNA datasets obtained from groups with high and low ImmuneScores. Differential gene expression was determined using the edgeR package in R, applying the criteria of p < 0.05 and | log_2_FC |> 1.

### Clustering analysis

Firstly, the above DEGs were selected, and their corresponding expression levels in each sample were extracted. Then, the ConsensusClusterPlus package [19] was used to perform cluster analysis on the samples, applying the parameter settings of ClusterAlg = "hc", Distance = "pearson", innerLinkage = "average", maxK = 2 and Reps = 10.

### Variation analysis

First, the maFtools package [20] was used to calculate the TMB corresponding to each TCGA-UCEC sample. Then, the differences in TMB among the above different clusters were compared, and the variation across different clusters was statistically analyzed.

### Prognostic analysis

Survival analysis was performed using Survival package [21]. Based on the above immunization scores and clustering results, survival analysis was performed and differences between groups were compared by log-rank test.

### Half maximal inhibitory concentration (IC_50_) analysis

According to the expression levels of cancer samples in RNA-seq and cGP2014 database [22], R package pRRophetic was used for IC_50_ analysis [23]. Then, the differences in IC_50_ scores among clusters were calculated using the Limma package.

### Immuno-infiltration analysis

Based on gene expression values, CIBERSORT (http://cibersort.stanford.edu/) was used to estimate the relative proportions of 22 immune cell types for each sample [24]. Next, survival analysis was performed on the relative proportions of the 22 immune cell types, and differences in immune cell proportions among clusters were examined using Wilcoxon's test.

### Analysis of gene set variation (GSVA)

The GSVA package [25] was utilized for GSVA analysis. Subsequently, enrichment scores were calculated for each gene set in each sample by using Wilcoxon's test, reflecting the relative activity of the gene sets across samples, as well as the differences in GSVA values for each item between clusters.

### Protein–protein interaction(PPI)

PPI analysis was performed by DEGs and plotted using Cytoscape with a screening criterion of combined_score > 0.4.

### Patients and clinical specimens

Fresh specimens from 20 EC patients were collected during surgery, with 10 normal tissues serving as controls. None of the patients had undergone anticancer chemotherapy, radiotherapy, drug treatment, or biological immunotherapy before surgery. Consent was obtained from all patients after providing them with written information, and a comprehensive patient profile can be found in Supplementary Table 1. Ethical approval for the study was granted by the Ethics Committee at Liuzhou Worker's Hospital, with the approval number KY2022300.

### Immunohistochemistry (IHC)

The 3 EC tissue specimens studied in this study were all formalin-fixed paraffin-embedded (FFPE) tumor blocks, and comprehensive clinical information for each sample was collected. The primary antibody targeting CTCF was sourced from Abcam (Ab128873, 1:1000, Abcam, Cambridge, UK). Subsequent to the addition of the primary antibody, an overnight incubation at 4 ℃ within a humidified container was conducted. Following the manufacturer's instructions (G1211, Servicebio). a three-step wash with phosphate-buffered saline (PBS) was performed, succeeded by treatment with a Histochemistry kit.

### Cell culture

The Chinese Academy of Sciences (Shanghai, China) provided us with 5 EC cell lines (AN3CA, HEC-1-B, RL95-2, HEC-1-A, and Ishikawa) and human endometrial endothelial cells (hEECs). The cell lines were cultured in RPMI-1640 medium with the addition of streptomycin (100 mg/mL), penicillin (100 U/mL), and 10% FBS (Gibco, USA). The culture was maintained at 37 ℃ in a humidified incubator with 5% CO_2_.

### Cell transfection

To down-regulate CTCF expression, 3 CTCF short hairpin RNA (sh-CTCF) and negative control (sh-NC) were designed and purchased from GeneChem (Shanghai, China), with their sequences listed in supplementary Table 2. Following the process of digestion and enumeration, EC cells (HEC-1-A and Ishikawa) were placed in six-well dishes with a concentration of 5 × 10^3^ cells per well and incubated for a duration of 12 h. When cell density reached 50%-70% of the total density of the culture dish, siRNA transfection was performed. The siRNA was first centrifuged for 1 min at 1000 rpm and dissolved in DEPC water (according to the product standard instruction), and then a pipette gun was used to resuspend it to mix thoroughly. Two combinations, Combination A (siRNA: 7.5 μL, opti-MEM: 180 μL) and Combination B (Lipo 2000: 7.5 μL, opti-MEM: 180 μL), were prepared individually and left undisturbed for a duration of 5 min. Subsequently, the two mixtures were combined to form Mixture AB. Following a 15 min incubation at ambient temperature, the cells were treated with Mixture AB. To maintain the culture, Mixture AB was thinned out in a full medium, and then a quantitative real-time polymerase chain reaction (qRT-PCR) assay was conducted to evaluate the transfection's effectiveness.

### qRT-PCR

Total mRNA was isolated from cells using the RNeasy Mini Kit (QIAGEN). Thermo Fisher's Superscript IV Reverse Transcriptase was employed for the reverse transcription of mRNA. PowerUp SYBR Green Master Mix (Thermo Fisher Scientific, USA) was utilized for conducting PCR reactions. The gene fold change was calculated using the 2^−ΔΔCt^ technique, normalizing against GAPDH as the control gene.

### TUNEL Staining

The evaluation of apoptosis in EC cells following various treatments was assessed using the one-step TUNEL apoptosis assay kit (TUNEL Apoptosis Assay Kit, Abcam, Cambridge, UK). Subsequently, the cells were cultured with DAPI (5 µg/mL) for nuclear staining. TUNEL-positive cells were observed using a light microscope (Axio Scope.A1, ZEISS, Germany). For the sake of robustness and reliability of the results, three wells were used in each treatment group, and the experiments were repeated three times.

### Transwell invasion assay

After a successful transfection, a total of 100000 cells were placed in each Transwell chamber (Corning, USA) that had been coated with 80 μL of Matrigel (1:8) and 100 μL of serum-free DMEM medium without serum. Complete medium was used to fill the lower Transwell chambers. After 24 h, the upper chambers were wiped with cotton swabs, and crystal violet staining was performed after 15 min to fix the successfully invaded cells on the lower Transwell chambers with 4% paraformaldehyde in a fixative solution. Using a microscope (Axio Observer Inverted Microscope, ZEISS, Germany), five randomly selected fields of view were photographed. For analysis purposes, the experiment was conducted thrice, and the mean value was computed.

### Wound healing assay

Following incubation in an incubator for 20 h in 6-well plates (with 3 × 10^5^ cells per well), HEC-1-A and Ishikawa cells were subjected to scratching using a pipette (10 μL). The removed cells were eliminated by rinsing with PBA. The collection of images took place at 0 h and 48 h, and the wound healing percentage was calculated using the following formula: (scratch healing area)/(original scratch area) × 100%.We repeated the experiment three times and calculated the average value.

### Subcutaneous tumor mouse model

Female BALB/c nude mice, aged six weeks, were acquired from the Shanghai Model Organisms Center, a company based in China. The mice were kept in animal facilities of SPF-grade, with a temperature of 24 ± 1 ℃, humidity between 50 and 60%, and a light/dark cycle of 12 h. The housing and experimental procedures followed the National Institutes of Health Guidelines for the Care and Utilization of Laboratory Animals. Approval for the research was granted by the animal ethics committee of Liuzhou Municipal Liutie Central Hospital, with the assigned approval number 2021-002-74. A total of 6 BALB/c nude mice were injected subcutaneously with around 10 million tumor cells (n = 6). Mice receiving cells transfected with sh-NC served as the control group (n = 6). Throughout the experiment, starting 8 days after tumor cell implantation, the weight of each mouse and the size of each tumor were measured using a vernier caliper every 4 days to monitor changes in tumor volume. The tumor volume was calculated by V = (W^2^ × L)/2, where V is tumor volume, W is tumor width, and L is tumor length. The mice were euthanized after 32 days.

### Flow cytometry analysis

First, tumor tissues from different groups of mice were collected and after cutting them into small pieces, the tissues were dissociated into single-cell suspensions using enzymatic digestion (collagenase and DNAase). Next, larger fragments were removed through filters and the cells were washed. Subsequently, erythrocytes were removed with erythrocyte lysate and the obtained single-cell suspension was subjected to cell counting. The cells were fixed with methanol and incubated with PE-conjugated CD49b and FITC-conjugated CD45. Finally, the percentage of natural killer (NK) cells was detected using a flow cytometer (Beckman Coulter, Life Sciences Division Headquarters, USA).

### Western blot

The BCA assay was used to determine the concentrations of proteins extracted from tumor tissue and cells. Initially, the proteins that were extracted were separated on gels made of SDS-PAGE and subsequently transferred to membranes made of PVDF. Following blocking with 5% skim milk, the proteins were incubated with primary antibodies overnight at 4 ℃ (CTCF antibody, 1:1,000, cat. no. ab128873; GAPDH antibody, 1:1,000, cat. no. ab8245; Abcam, Cambridge, UK). The next day, HRP-conjugated secondary antibodies (1:2,000, #ab205718, Abcam, Cambridge, UK) were added for subsequent incubation. The fluorescence imager (Alpha) was used for visualization, and the levels of specific proteins were normalized to the levels of GAPDH.

### Statistical analysis

Microsoft Excel, GraphPad Prism 8.0, and R (version 4.1.0) were utilized for performing statistical analyses and generating visualizations. For normally distributed data, results were usually expressed as mean ± standard deviation (mean ± SD), and t-tests (two-group comparisons) or one-way ANOVA (multiple comparisons) were used to test for differences between groups. For analyses involving two independent variables, such as treatment groups and time points, two-way ANOVA was performed to evaluate the effects of both factors and their interaction on tumor volume or other outcomes. Clinical characterization reports use different methods of statistical description depending on the type of data, the average standard deviation or n (%). Statistical significance was determined by p ≤ 0.05, unless stated otherwise, and the Benjamini–Hochberg method was used to correct for multiple testing [26].

## Results

### ImmuneScores of the 543 samples and survival analysis

**Fig. 1:**
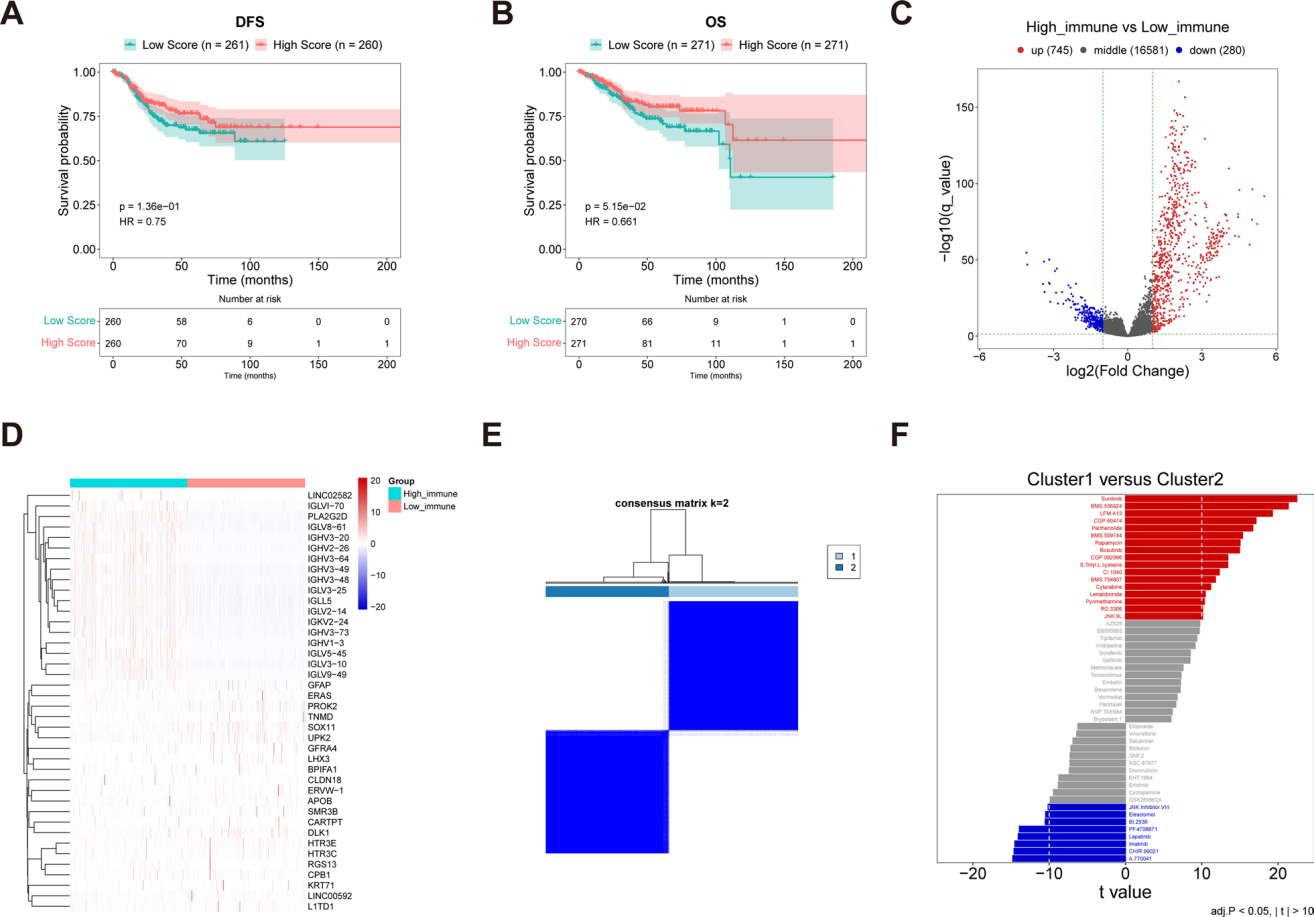
Comparative analysis of survival and GSVA between groups with high scores and groups with low scores. **A** Survival analysis in DFS. **B** Survival analysis in OS. **C** Volcano plot. **D** Heat map. **E** Consensus matrix. **F** GSVA score map

The RNA-seq data from 543 EC samples were acquired from the GDC data portal and analyzed using the ESTIMATE R package. The estimation results for each sample, which include StromalScore, ImmuneScore, tumor purity, and ESTIMATEScore, are presented in supplementary Table 3. By employing immune scores, the 543 samples were categorized into groups with high and low scores. Subsequently, survival analysis was performed using the UCEC database. The log-rank test (Fig. 1A, B) was used to calculate the p-values for disease-free survival (DFS) and overall survival (OS). Significant differences (p = 0.136) were not found in the comparison between high and low-score groups in DFS. But OS was higher in patients with high immunization scores than in those with low immunization scores (p = 5.15e-02).It reveals that there are key molecules or pathways involved in tumor progression or suppression at different levels of immune infiltration.

### Comparison of DEGs and GSVA between groups with high scores and groups with low scores

Differential gene expression was determined using the edgeR package in R, applying the criteria of p < 0.05 and |log_2_FC |> 1. Among the 1025 DEGs identified, there are 745 genes that were up-regulated and 280 genes that were down-regulated in the high scores group compared to the low scores group, as depicted in Fig. 1C and Supplementary Table 4. Next, we used heat maps to analyze DEGs based on explicit DEG groups and immunity scores. Notably, 20 genes exhibited both high expression levels and high ImmuneScores, including LINC02582, IGLVI-70, PLA2G2D, and IGLV8-61, while genes such as GFAP, ERAS, PROK2, TNMD, and SOX11 displayed the opposite expression pattern (Fig. 1D).

Afterwards, the samples were grouped into Cluster 1 and Cluster 2, taking into account the 1025 DEGs that distinguished the high and low ImmuneScore groups (Fig. 1E). The pRRophetic package was utilized to conduct IC_50_ analysis, which was based on the gene expression matrix of clusters 1 and 2. Notably, A.770041, CHIR.99021, Imatinib, BI.2536, and JNK.Inhibitor.VIII played crucial roles in cluster 1, while Sunitinib, BMS.536924, LFM.A13, CGP.60474, Parthenolide, BMS.509744, Rapamycin, Bosutinib, CGP.082996, S.Trityl.L.cysteine, CI.1040, BMS.754807, Cytarabine, Pyrimethamine, and JNK.9L showed significant correlations with samples in cluster 2 (Fig. 1F).

Furthermore, utilizing datasets in Hallmark and the GSEA R package with the ssGSEA method, GSVA was performed. A wilcox.test was employed to compare the two clusters. Significantly enriched Kyoto Encyclopedia of Genes and Genomes (KEGG) terms in cluster 1 were primarily related to adipogenesis, peroxisome, glycolysis, and hedgehog signaling. On the other hand, cluster 2 exhibited an abundance of terms related to signaling, heme metabolism, PI3K activation, AKT/mTOR signaling, and IL-6/JAK/STAT3 signaling (Supplementary Fig. 1).

### Genes mutation frequency analysis between cluster1 and cluster2

**Fig. 2:**
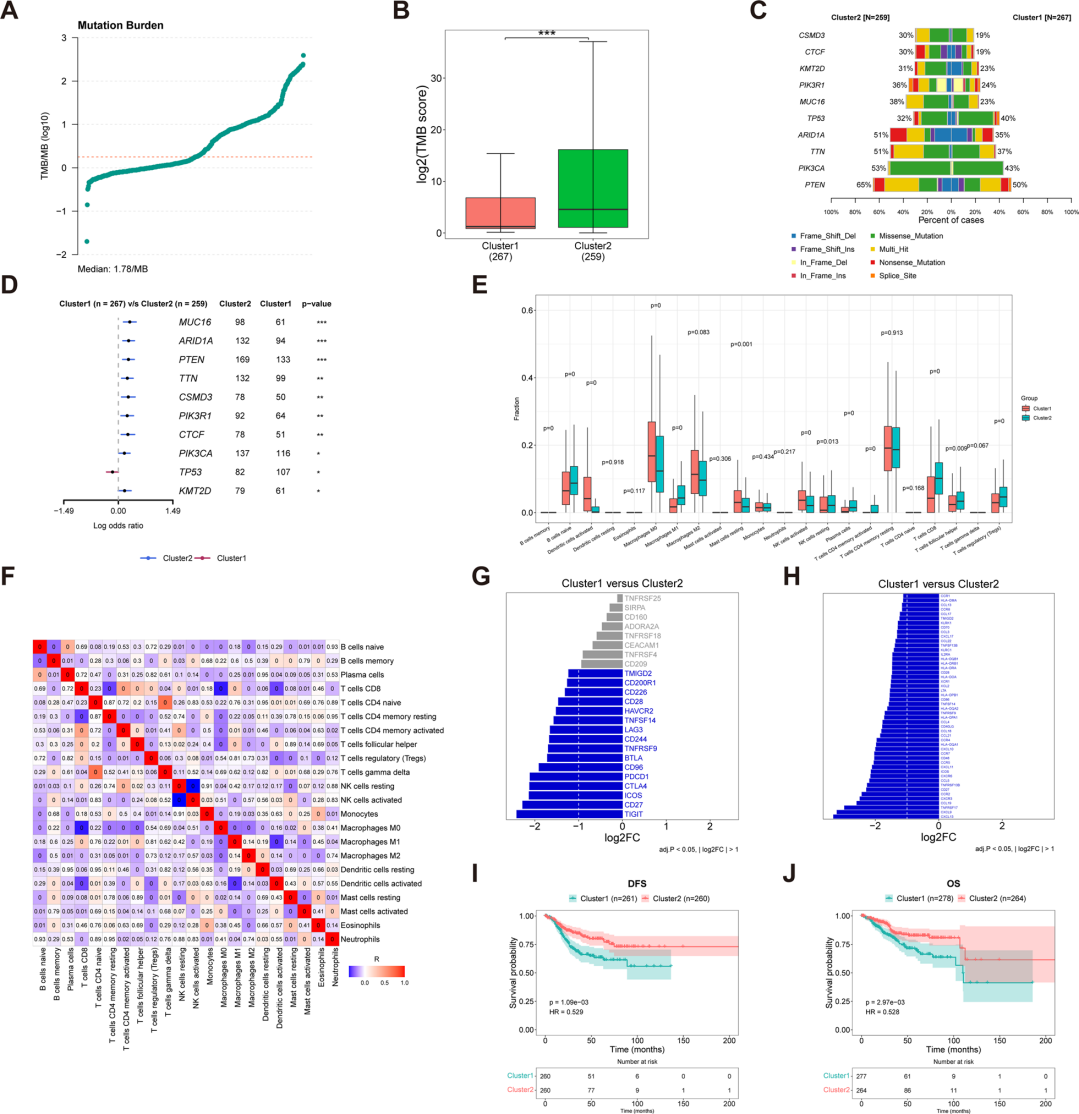
The analysis of TMB for the genes and the analysis of infiltrating immune cells were conducted between the two clusters. **A** Mutation burden. **B** TMB score. **C** Percentage of cases. **D** Log odds ratio. **E** significantly enriched immune cells. **F** Correlation analysis of the immune cells. **G** Differentially expressed immune checkpoints. **H** Differentially expressed immune regulatory factors. **I** Survival analysis in DFS. **J** Survival analysis in OS

To assess the mutation frequency of genes in Cluster 1 and Cluster 2, Maftools was employed to calculate the TMB, representing the number of replacements and insertions/deletions per megabyte in the gene's exon coding region. In Fig. 2A, the TMB scores showed a notable divergence between Cluster 1 and Cluster 2 (p < 0.001, Fig. 2B). The top ten genes with the highest mutation frequency were then analyzed, presenting the percentage of cases and log odds ratio (Fig. 2C, D). Among these genes, MUC16, ARID1A, PTEN, TTN, CSMD3, PIK3R1, CTCF, PIK3CA, and KMT2D exhibited more mutation sites in Cluster 2. Interestingly, the gene TP53 demonstrated more mutation sites in Cluster 1.

### Infiltrating immune cells analysis

In addition to tumor cells, different types of cells in the tumor microenvironment play a role in promoting tumor growth. The evaluation of progress and potential response to immunotherapy becomes crucial by identifying infiltrating immune cells, based on the idea of cold and hot tumors. The CIBERSORT web tools were utilized to analyze immune cells, uncovering notable abundance of inexperienced B cells, M1 macrophages, inactive NK cells, plasma cells, CD4⁺ T memory cells T cells, CD8⁺ T cells, T follicular helper cells, and regulatory T cells (Tregs) in both Cluster 1 and Cluster 2 (Fig. 2E). The correlation analysis revealed strong correlations among these immune cells, including gamma delta T cells, naive CD4⁺ T cells, and plasma cells (Fig. 2F). Differences in immune checkpoints and immune regulatory factors between clusters were calculated using the Limma package, revealing distinct expressions of 16 immune checkpoints (e.g., TIGIT, CD27, ICOS, CTLA4) and 50 immune regulatory factors (e.g., CXCL13, CXCL9, TNFRSF17, CCL19) between Cluster 1 and Cluster 2 (Fig. 2G, H).

Survival differences among different clusters were compared using the log-rank test based on clinical data and clustering conditions during the conduction of survival analysis. Cluster 2 exhibited significantly higher survival rates compared to Cluster 1 (Fig. 2I, J).

### CTCF was up-regulated in EC

**Fig. 3:**
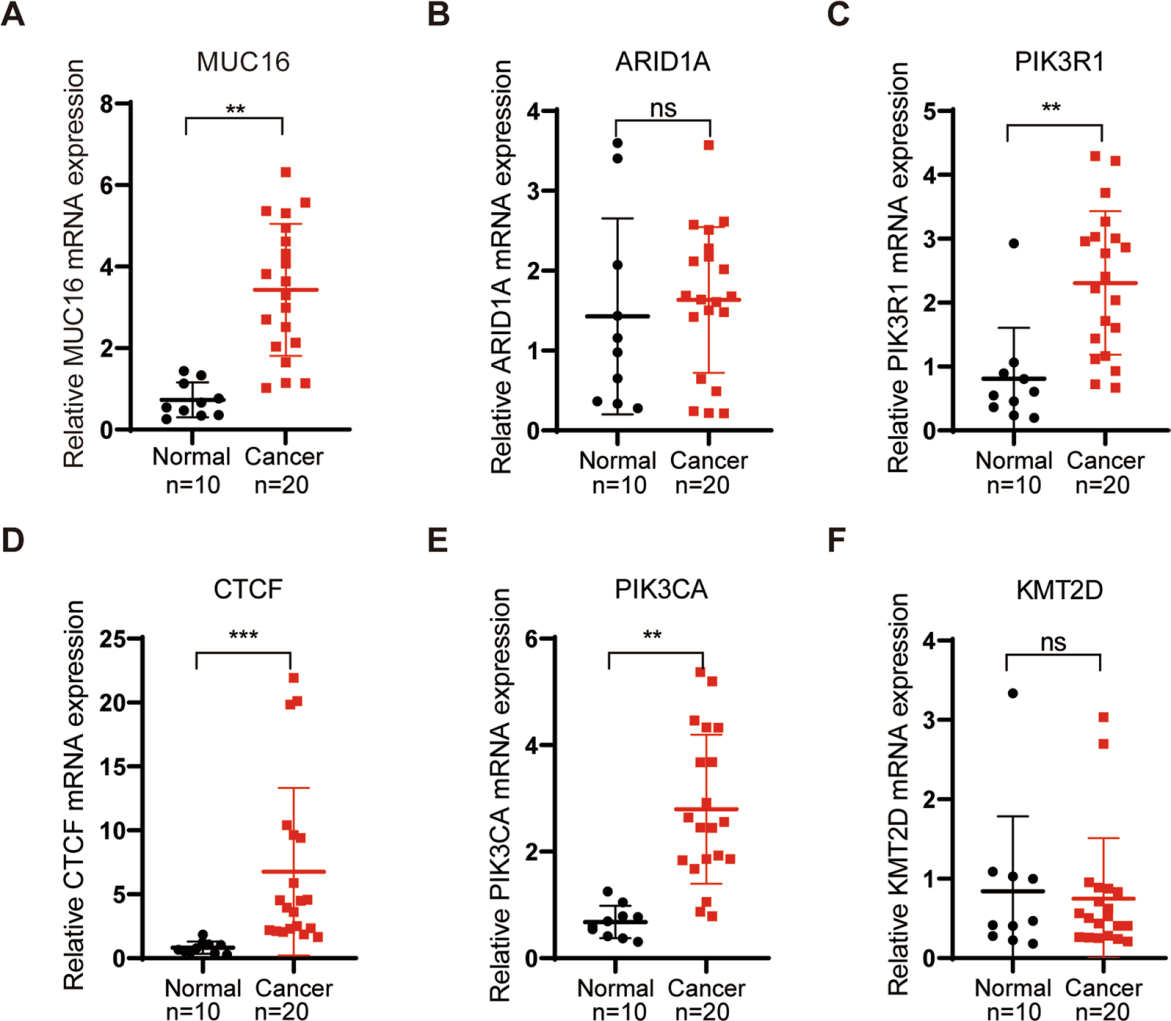
Validation of expression of representative DEGs in clinical samples. qRT-PCR assay was performed to validate the expression of selected DEGs in EC samples and tumor-adjacent samples. Normal: n = 10; Cancer: n = 20.** A** MUC16 was up-regulated in EC. **B** ARID1A expression exhibited no significant difference. **C** PIK3R1 was up-regulated in EC. **D** CTCF was up-regulated in EC. **E** PIK3CA was up-regulated in EC. **F** There was no notable variation in the expression of KMT2D."ns" indicates non-significant, ^**^p < 0.01, ^***^p < 0.001

The expression of representative DEGs from bioinformatics analysis was further validated in EC clinical samples. There was significant overexpression of mRNA for MUC16, CTCF, PIK3R1, and PIK3CA in the EC samples as compared with normal tissues (Fig. 3A and C–E), while no significant difference was observed for ARID1A and KMT2D (Fig. 3B and F). Considering that CTCF was the most significantly expressed, it was chosen for further study.

**Fig. 4:**
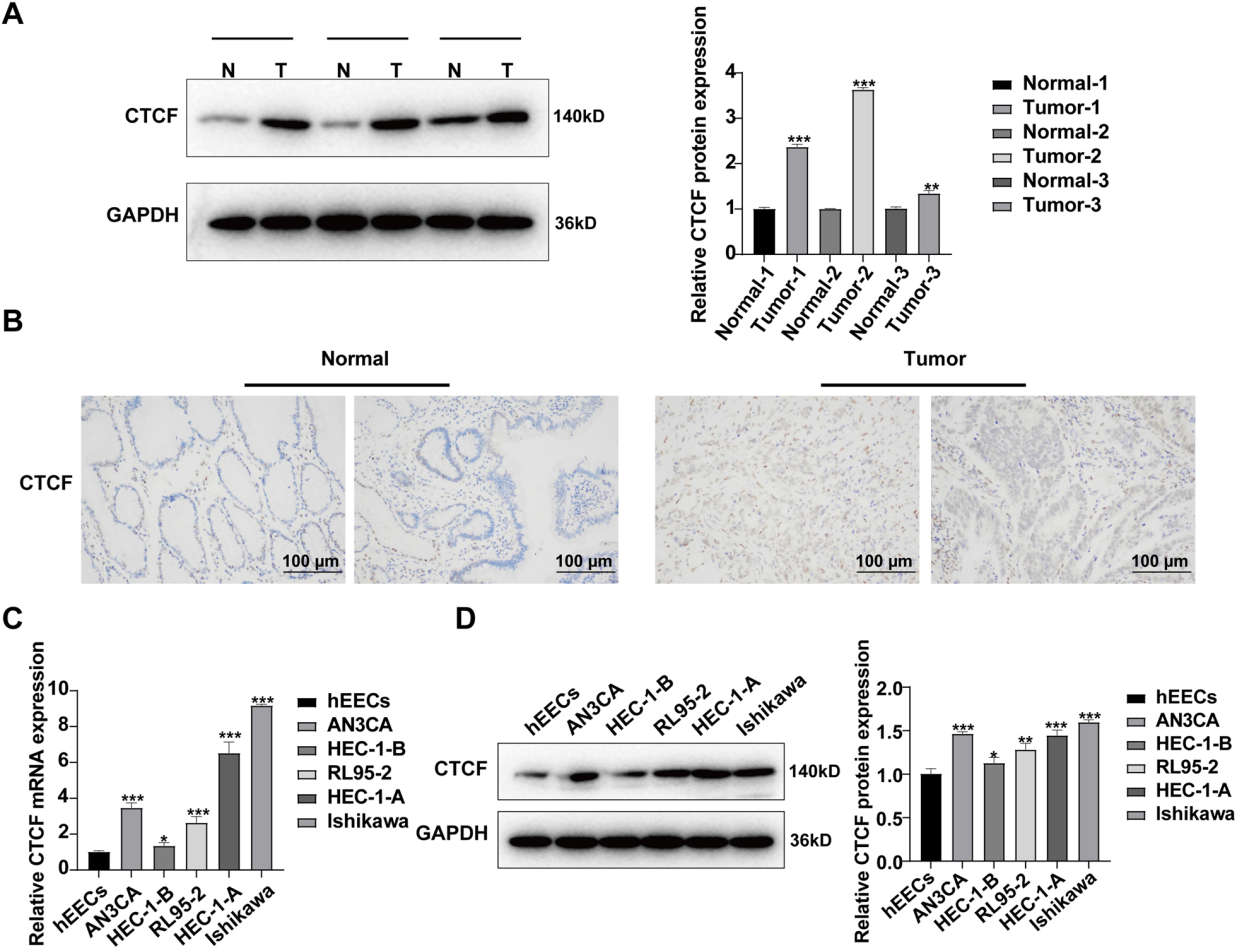
CTCF was up-regulated in EC clinical samples and cell lines. **A** WB assay and **B** IHC assay were performed to determine the expression of CTCF in EC clinical samples (Normal: n = 3; Tumor: n = 3). **C** qRT-PCR assay and **D** WB assay were performed to validate the expression of CTCF in EC cell lines. CTCF was up-regulated in 5 EC cell lines compared with normal human endometrial endothelial cells. n = 3 biologically independentsamples. ^*^p < *0.05,* ^**^p < 0.01, ^***^p < 0.001

Next, CTCF protein expression was detected in EC clinical samples and cells. CTCF protein showed significantly up-regulated expression in both 3 EC clinical samples and 5 EC cell lines (Fig. 4A–D).

### Suppression of CTCF hindered the migratory and invasive capabilities of EC cells

**Fig. 5:**
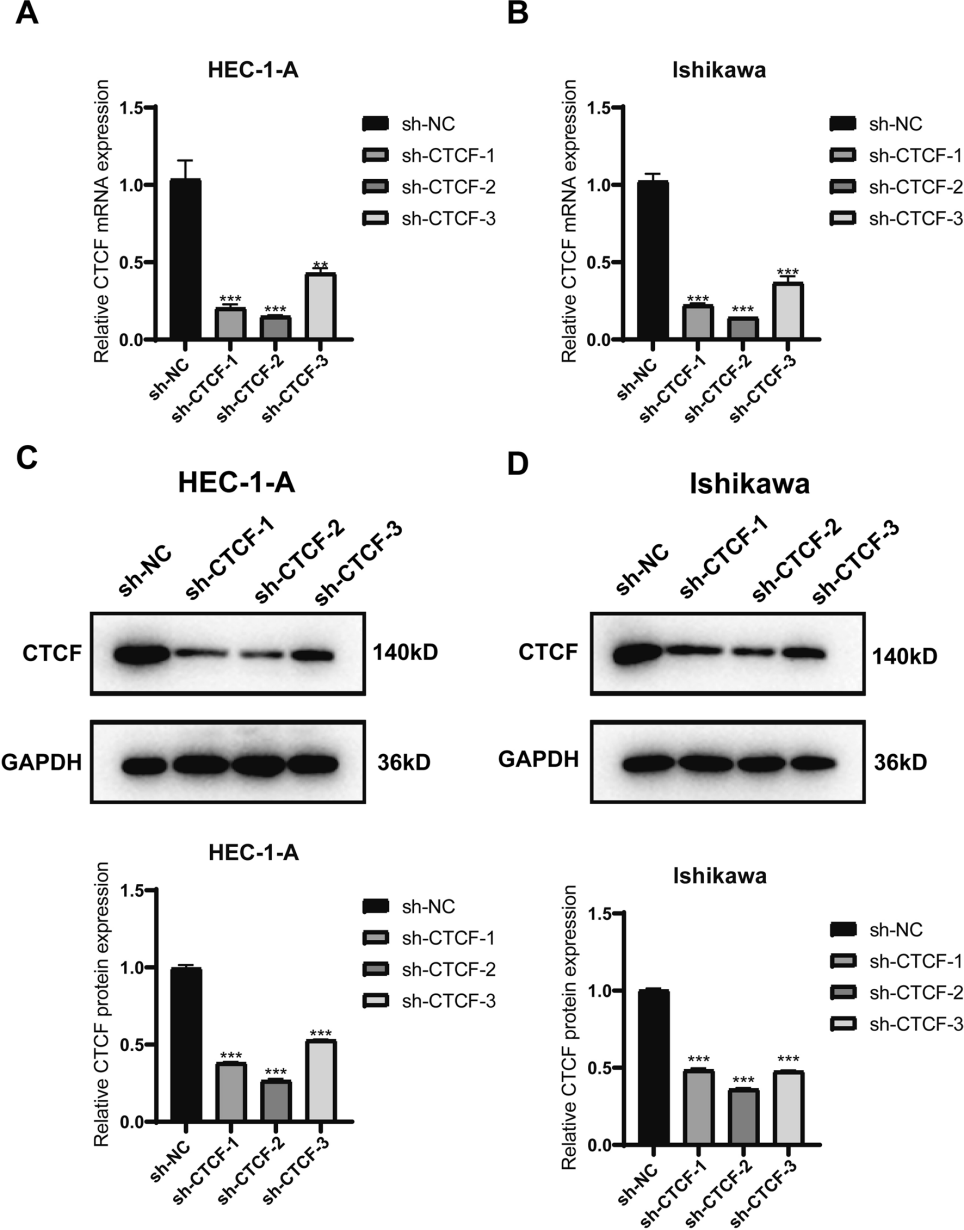
shCTCF-2 exhibited the best efficiency in lowering CTCF expression. qRT-PCR was used to detect the CTCF expression after knocking down CTCF in **A** HEC-1-A and **B** Ishikawa cell line. WB assay was used to detect the CTCF expression after knocking down CTCF in **C** HEC-1-A and **D** Ishikawa cell line. n = 3 biologically independentsamples. ^**^ p < 0.01, ^***^p < 0.001

To further explore the role of CTCF in EC, we next assessed the function of CTCF in cell invasion and migration. Among the 3 sh-CTCFs, sh-CTCF-2 demonstrated the most significant downregulation in CTCF expression (Fig. 5A–D). Thus, we subsequently chose sh-CTCF-2 for knockdown experiments.

**Fig. 6:**
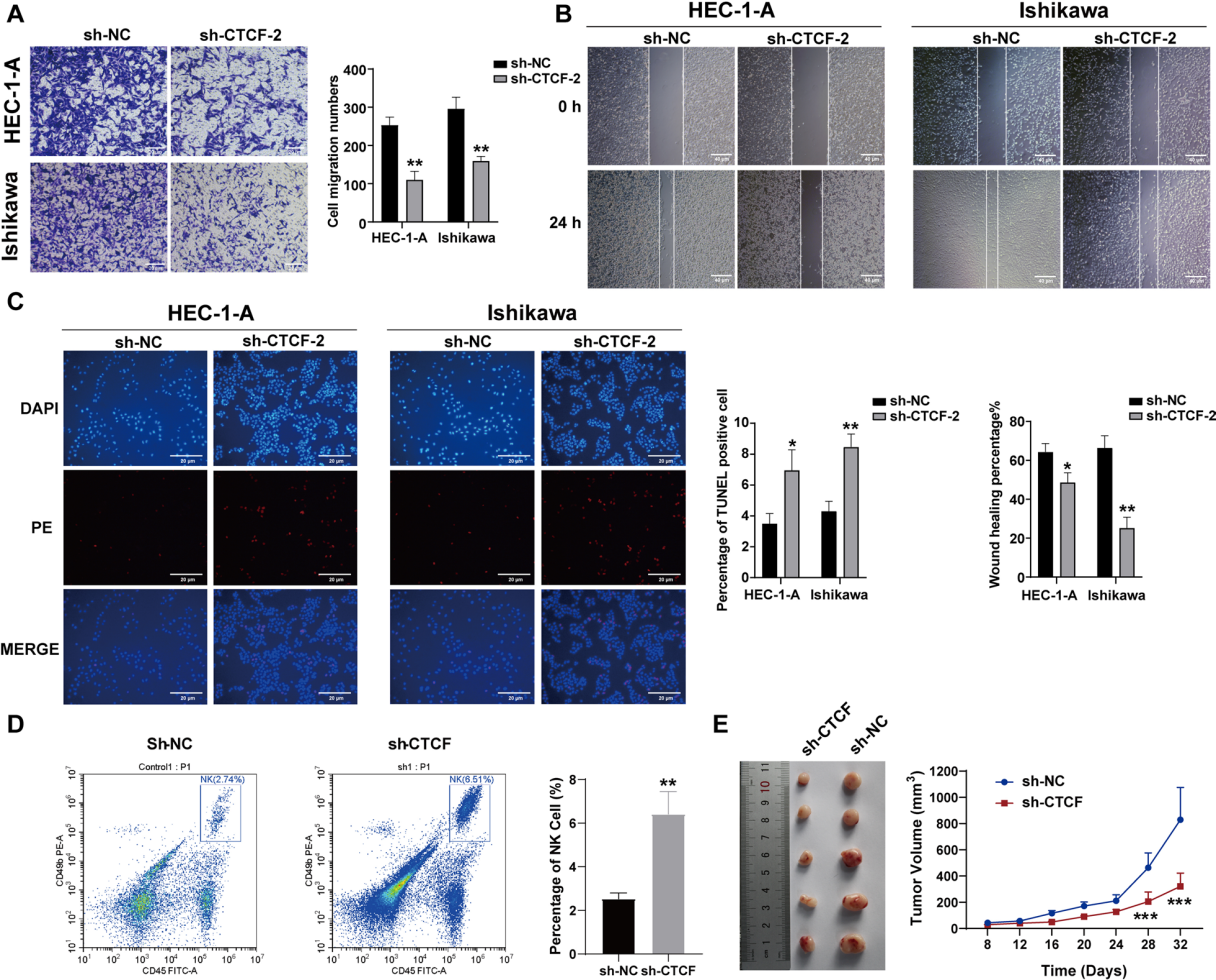
Effect of CTCF gene knockdown on EC cells. Knocking down CTCF inhibited **A** invasion and **B** metastasis ability of HEC-1-A and Ishikawa cell lines. **C** Knocking down CTCF promoted HEC-1-A and Ishikawa cell apoptosis. **D** Knocking down CTCF promoted HEC-1-A percentage of NK cell. Each experiment was repeated three times. **E** Knocking down CTCF significantly inhibited tumor growth (n = 5, One mouse out of six was not included in the final count because it did not meet the preset experimental criteria and died.). ^*^p < 0.05, ^**^p < 0.01, ^***^p < 0.001

Transwell and cell scratch assays showed that knockdown of CTCF significantly attenuated the invasive and metastatic abilities of EC cells (Fig. 6A–B). Moreover, decreased CTCF expression also promotes apoptosis in EC cells (Fig. 6C). To assess the impact of CTCF knockdown on tumor growth, we established a subcutaneous EC model in thymus-free nude mice using EC cells stably transfected with sh-NC and sh-CTCF (Supplementary Fig. 2). Since a significant cluster of immune cells, including NK cells, was identified in the infiltrating immune cell analysis section, we tested the effect of knocking down CTCF on NK cell activity in mice in vivo. Notably, sh-CTCF significantly increased the proportion of NK cells and exhibited a pronounced inhibitory effect on tumor growth. (Fig. 6D–E).

### Analysis of the potential mechanism of action of CTCF in EC

**Fig. 7:**
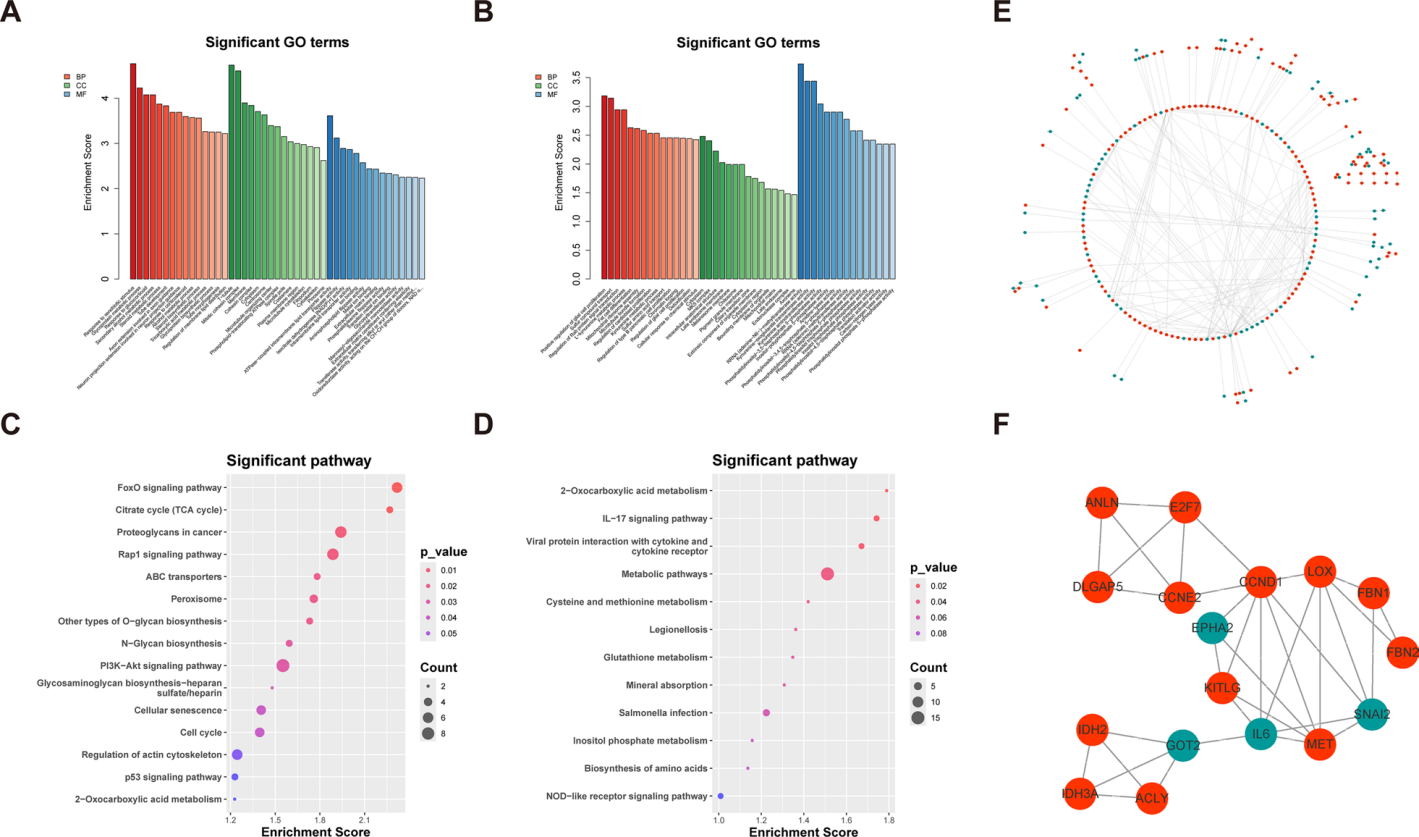
Analysis of the potential mechanism of action of CTCF in EC. **A** GO enrichment analysis of up-regulated DEGs **B** GO enrichment analysis of down-regulated DEGs. **C** KEGG enrichment analysis of up-regulated DEGs. **D** KEGG enrichment analysis of down-regulated DEGs. **E** PPI analysis. **F** Subnetwork diagram of hub genes in E

To deeply investigate the mechanism of CTCF action in EC, we performed Gene Ontology (GO) and KEGG pathway enrichment analysis. Experimental data related to CTCF genes were found through the online database GSPAdb [27] (https://www.gpsadb.com/). After interfering (including shRNA, siRNA, KO, etc.) with CTCF gene expression, the downstream genes of CTCF were obtained, and the screening criteria were FC > 1.5 and p < 0.05. The dataset information is in Table S5. GO enrichment analysis showed that up-regulated DEGs were significantly enriched in glucose metabolism, steroid metabolic process, and ATP transport (Fig. 7A, Table S6), and down-regulated DEGs were significantly enriched in the regulation of Rab protein signaling, L-kynurenine metabolism process, and rRNA (adenine-N6-)-methyltransferase_activity(Fig. 7B, Table S7). KEGG enrichment analysis also showed enrichment of multiple metabolism-related pathways, with up-regulated DEGs enriched in the tricarboxylic acid cycle and signaling pathways such as FoxO, Rap1, and PI3K-Akt (Fig. 7C, Table S8), while down-regulated DEGs were mainly enriched in the 2-Oxocarboxylic acid metabolism and IL-17 signaling pathway (Fig. 7D, Table S9). It is suggested that CTCF may contribute to EC progression by affecting the metabolic pathways of EC cells and thus promoting EC progression.

Next, based on the obtained differential genes, we performed PPI analysis by STRING database, obtained hub genes based on Degree, selected top10 as hub genes, and mapped the network using Cytoscape. The results showed that CTCF was significantly regulated with IL6, MET, LOX, CCND1 (Fig. 7E–F). A related study reported that both CCND1 and CTCF were identified as significant mutated genes in cervical cancer[28]. This suggests that CCND1 may be a potential regulatory factor of CTCF, collectively influencing the progression of EC, which points to the direction for our next research. Our future studies will further investigate the in-depth mechanism of CCND1 and CTCF in EC and provide new ideas for the clinical treatment of EC.

## Discussion

EC is among the most common tumors of the female reproductive tract [29]. Given that active and appropriate treatment can effectively prolong the survival of patients, accurate prognosis assessment is of great significance for the rational selection of treatment [30, 31]. TCGA project, combining immune characteristics and molecular changes, has conducted a more refined classification of EC patients, enabling clinical treatments to be more precise and personalized, which is of significant importance to clinicians [32, 33]. The present study retrospectively analyzed 543 cases of EC, conducted follow-ups, explored factors influencing the prognosis based on precise classification, and explored potential treatment targets. After categorizing 543 samples into groups based on ImmuneScores, it was observed that a higher score was associated with a more favorable prognosis. Then, DEGs were identified, and EC patients were divided into two clusters. TMB analysis showed that genes in cluster 2 possessed a higher mutation rate, such as MUC16, ARID1A, PTEN, TTN, CSMD3, PIK3R1, CTCF, PIK3CA, and KMT2D. These genes have been validated to be associated with EC in other studies [34, 35]. MUC16 is a serum marker for EC diagnosis and prognosis [36]. Besides, Akt phosphorylation after the loss of PTEN expression represents a poor prognostic factor in EC [37]. Further survival analysis suggested that compared to cluster 1, cluster 2 correlated with significantly higher survival rates.

Among the above highly-mutated genes, we validated their expression in clinical EC samples and cell lines, and CTCF showed the most significant differential expression. CTCF is a nuclear protein containing 11 contiguous zinc finger domains. It has a variety of intracellular functions, including transcriptional activation, transcriptional repression, X chromosome inactivation, gene imprinting, and regulation of the three-dimensional conformation of chromatin [38]. In cancer, CTCF can play both oncogenic and anti-tumor roles. Specifically, CTCF can bind to the promoter region of the tumor suppressor gene P53 to inhibit the enrichment of chromatin repression markers such as H3K9me3, H3K27me3, and H4K20me3, thereby maintaining the transcriptional activity of P53 and exerting a tumor suppressor function in various tumor cells [39]. In human breast cancer cells, CTCF can inhibit the expression of the pro-apoptotic protein Bax, thereby exerting an anti-apoptotic effect [40]. However, the role of CTCF in EC is not fully clear. A recent study revealed that CTCF mutation in EC could promote cell survival and correlate with EC recurrence and metastasis [41]. However, other studies have suggested that CTCF has a suppressive role in EC. Compared to WT mice, tumors in heterozygous CTCF mice exhibit higher invasiveness in terms of invasion, metastatic spread, and mixed epithelial/stromal differentiation, confirming CTCF as a haploinsufficient tumor suppressor [42]. The present study showed that CTCF knockdown could significantly restrain EC cells from migrating and invading and inhibited tumor growth in EC-bearing mice, indicating CTCF may play an anti-tumor effect in EC.

Moreover, we also analyzed the immune landscape in EC by examining the infiltration of different immune cells. Naive B cells, M1 macrophages, resting NK cells, CD4⁺ memory T cells, CD8⁺ T, and others were enriched in cluster 1 and cluster 2. Besides, immune checkpoints and immune regulatory factors showed different distributions in the two clusters. As a potential key molecule for the anti-tumor effect following anti-CTLA-4 therapy [43], ICOS exhibited a fourfold change in expression between Cluster 1 and Cluster 2 in our study. Other immune checkpoints, such as CTLA4, CD96, and BTLA, also showed significant changes, consistent with findings from other studies [44]. The field of cancer immunotherapy is progressively shifting its attention towards TIGIT, which is a suppressive receptor found on lymphocytes [45]. According to Harjunpää and Guillerey [46], there are reports suggesting that TIGIT has the ability to hinder various stages of the cancer immunity cycle, which includes suppressing anti-tumor reactions. In the present study, TIGIT was differentially expressed between the groups based on the ImmuneScores. Another study revealed that artesunate-induced ATG5-related autophagy could enhance NK92 cell cytotoxicity onEC cells by interacting with CD155 and CD226/TIGIT [47]. Interestingly, the differential expression of CD226 was also identified in our research, indicating its potential role in predicting EC.

The findings from our study hold promising implications for potential clinical applications. The identification of key genetic markers, such as CTCF, associated with prognosis and treatment response in EC suggests the feasibility of targeted therapies tailored to specific molecular subtypes. The observed differences in immune cell infiltration and checkpoint expression between distinct clusters may guide the development of immunotherapeutic strategies. Furthermore, the correlation of TMB with specific genes and their association with survival rates highlights the potential for incorporating mutational burden assessments into clinical decision-making processes. However, there still are some limitations in our study. Firstly, the sample size of 543 cases, while substantial, may not capture the full spectrum of molecular and clinical heterogeneity within EC. The focus on specific genetic markers, such as CTCF, may overlook other potentially relevant molecular factors contributing to the complexity of EC. Second, for the investigation of the mechanism of action of CTCF, a large number of experiments are still needed to analyze the effects of CTCF and CCND1 on EC. Furthermore, the generalizability of our findings to broader populations may be influenced by the specific characteristics of the patient cohort studied. The dynamic nature of immune responses and the tumor microenvironment presents challenges in interpreting the observed ImmuneScores, and further investigations are warranted to elucidate the temporal dynamics of these interactions. This study found that si-CTCF significantly reduced tumor tissue size, suggesting a potential relationship with TIICs and their interactions. However, the specific regulatory mechanisms still require further verification [48]. In spite of these constraints, our investigation offers valuable perspectives on the molecular and immune characteristics of EC, establishing a foundation for forthcoming research efforts focused on tackling these obstacles.

## Conclusions

Herein, we explored the molecular typing characteristics of EC based on high-throughput data mining and preliminarily constructed a gene molecular typing model for EC prognosis. Knockdown of CTCF could decrease the migration ability and invasion ability of EC cells and promote EC apoptosis in EC cells. Furthermore, knocking down CTCF could inhibit tumor growth in vivo. And we found that CTCF may promote EC progression by affecting the metabolic pathways of EC cells through KO and KEGG enrichment analysis. Moreover, potential regulators of CTCF were identified by PPI analysis.In general, our discoveries offer new perspectives on the molecular process of EC.

## Supplementary Information

Additional file 1: Figure 1. Significantly enriched KEGG terms between cluster 1 and cluster 2.

Additional file 2: Figure 2. Knocking down CTCF inhibited tumor growth.

Additional file 3.

Additional file 4.

Additional file 5.

Additional file 6.

Additional file 7.

Additional file 8.

Additional file 9.

Additional file 10.

## Data Availability

Data is provided within the manuscript or supplementary information files.

## Abbreviations

EC: Endometrial cancer
GO: Gene ontology
KEGG: Kyoto encyclopedia of genes and genomes
TMB: Tumor mutation burden
DEGs: Differentially expressed genes
qRT-PCR: Quantitative real-time polymerase chain reaction
IHC: Immunohistochemistry
NK: Natural killer
TCGA: The cancer genome atlas
PPI: Protein–protein interaction
IC_50_: Half maximal inhibitory concentration
