# Supplementary figures and images for: Immune alterations and overexpression of CTCF in endometrial carcinoma: insights from molecular subtyping
Source: Cancer Cell Int. 2024 Dec 2;24:392. doi: 10.1186/s12935-024-03576-y (PMC11613940; doi:10.1186/s12935-024-03576-y)

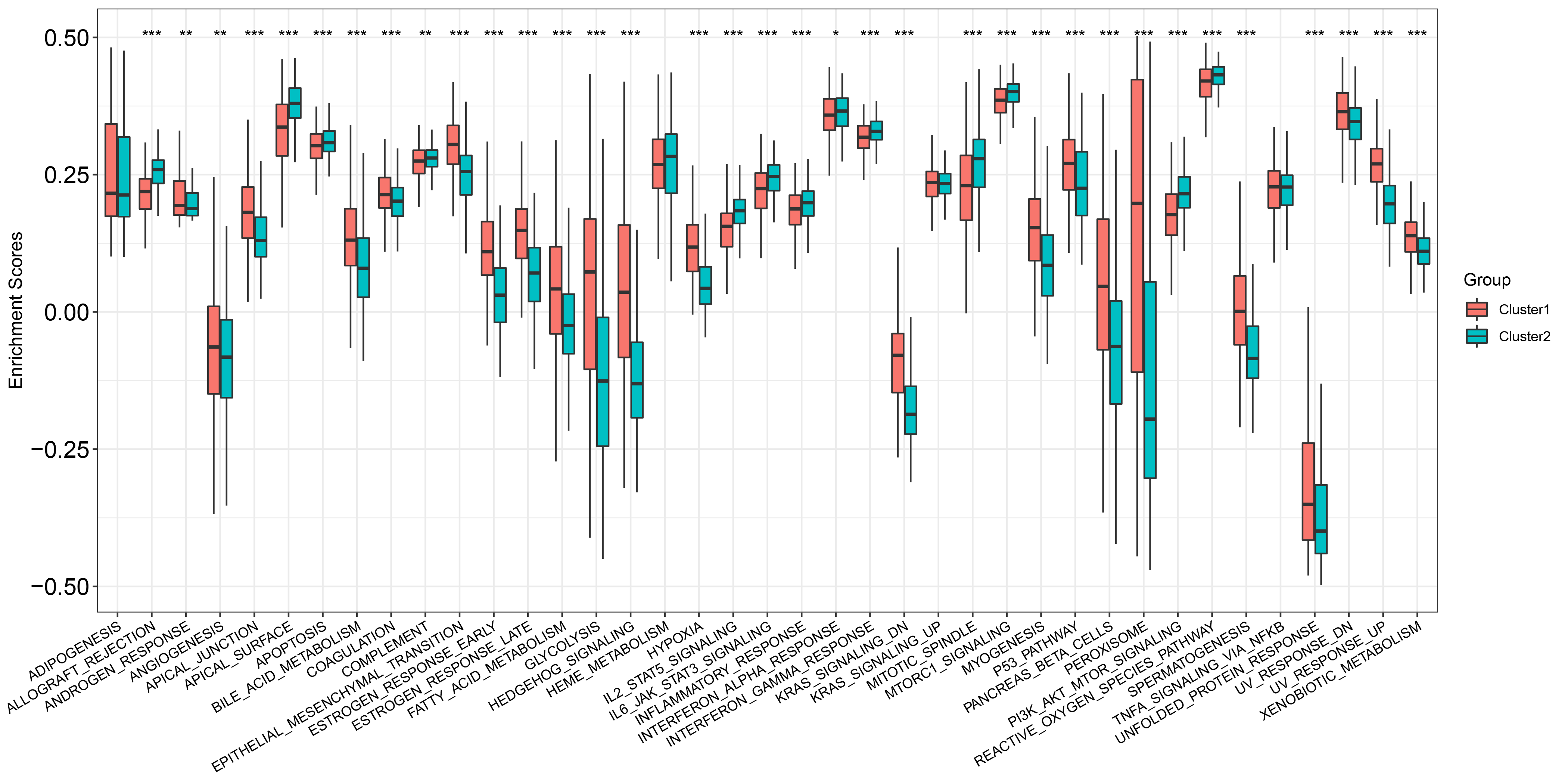

Supplement: Supplementary file 1 — Additional file 1: Figure 1. Significantly enriched KEGG terms between cluster 1 and cluster 2. [file 12935_2024_3576_MOESM1_ESM.tif]

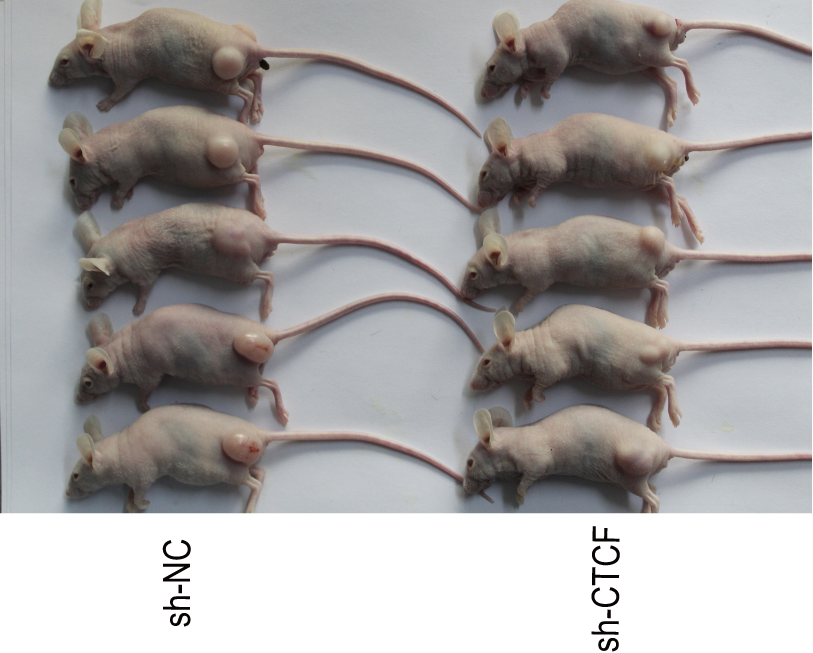

Supplement: Supplementary file 2 — Additional file 2: Figure 2. Knocking down CTCF inhibited tumor growth. [file 12935_2024_3576_MOESM2_ESM.tif]
