# Supplementary material for: Immune alterations and overexpression of CTCF in endometrial carcinoma: insights from molecular subtyping
Source: Cancer Cell Int. 2024 Dec 2;24:392. doi: 10.1186/s12935-024-03576-y (PMC11613940; doi:10.1186/s12935-024-03576-y)
Supplement: Supplementary file 3 — Additional file 3. [file 12935_2024_3576_MOESM3_ESM.doc]

**Table S1. Clinical information**

|  | **Gender** | **Age** | **Tissue types collected** | **Pathological Pattern** | **Stage** | **Differentiated degree** | **pTNM stage** | **FIGO stage** | **Tumor Size** |
| --- | --- | --- | --- | --- | --- | --- | --- | --- | --- |
| **1** | Female | 60 | **cancer** | Endometrioid adenocarcinoma | G3 | Low | T3aN2M0 | IIIC2 | 11×8.5×4cm |
| **2** | Female | 48 | **cancer** | Endometrioid adenocarcinoma | G1 | High | T1aN0M0 | IA | 6×4×1.8cm |
| **3** | Female | 50 | **cancer** | Endometrioid adenocarcinoma | G3 | Low | T2N0M0 | II | 9×5×2.5cm |
| **4** | Female | 47 | **cancer** | Endometrioid adenocarcinoma | G2 | Medium | T1aN0M0 | IA | 3×2.5×1cm |
| **5** | Female | 60 | **cancer,normal(adjacent)** | Endometrioid adenocarcinoma | G3 | Low | T1aN0M0 | IA | 4.5×3.6×2.5cm |
| **6** | Female | 47 | **cancer,normal(adjacent)** | Endometrioid adenocarcinoma | G2 | Medium | T1aN0M0 | IA | 5×4×1.5cm |
| **7** | Female | 57 | **cancer,normal(adjacent)** | Endometrioid adenocarcinoma | G2 | Medium | T1aN0M0 | IA | 6.5×6.5×1.5cm |
| **8** | Female | 54 | **cancer** | Endometrioid adenocarcinoma | G2 | Medium | T1aN0M0 | IA | 6.3×4×1.2cm |
| **9** | Female | 60 | **cancer,normal(adjacent)** | Endometrioid adenocarcinoma | G3 | Low | T2N0M0 | II | 7.5×7.5×2cm |
| **10** | Female | 49 | **cancer,normal(adjacent)** | Poorly differentiated carcinoma combined with endometrioid carcinoma grade Ⅱ | G3+G2 | Low+Medium | T4N1M1 | IVB | 6.7×5×3cm |
| **11** | Female | 58 | **cancer** | Endometrioid carcinoma combined with undifferentiated carcinoma | G2 | Medium | T2N0M0 | II | 4.6×3.5×2.8cm |
| **12** | Female | 53 | **cancer,normal(adjacent)** | Endometrioid adenocarcinoma | G2 | Medium | T1aN0M0 | IA | 8×5.5×2.5cm |
| **13** | Female | 63 | **cancer** | Endometrioid adenocarcinoma | G2 | Medium | T1aN0M0 | IA | 4cm×3cm |
| **14** | Female | 53 | **cancer,normal(adjacent)** | Endometrioid adenocarcinoma | G2 | Medium | T1aN0M0 | IA | 4×1.8cm |
| **15** | Female | 66 | **cancer,normal(adjacent)** | Endometrioid adenocarcinoma | G2 | Medium | T1aN0M0 | IA | 6×6×2cm |
| **16** | Female | 59 | **cancer** | Endometrioid adenocarcinoma | G3 | Low | T2N0M0 | II | 5.5×4.5×1.6cm |
| **17** | Female | 50 | **cancer,normal(adjacent)** | Endometrioid adenocarcinoma | G1 | High | T1aN0M0 | IA | 4.5cm×4cm×0.5cm |
| **18** | Female | 72 | **cancer** | Endometrioid adenocarcinoma | G2 | Medium | T1bN0M0 | IB | 2cm×1.7cm×1.5cm |
| **19** | Female | 56 | **cancer,normal(adjacent)** | Endometrioid adenocarcinoma | G2 | Medium | T2N0M0 | II | 1.9×1.6×0.4cm |
| **20** | Female | 52 | **cancer** | Endometrioid adenocarcinoma | G3 | Low | T1aN1M0 | IIIC1 | 5×3cm |

**Table S2. The sequences of CTCF short hairpin RNA (shCTCF) and negative control (shNC).**

| **Names** | **DNA Sequences for short hairpin RNA** |
| --- | --- |
| shCTCF-1 | 5’-GCGGAAAGTGAACCCATGATA-3’ |
| shCTCF-2 | 5’-GCAAGGCAAGAAATGCCGTTA-3’ |
| shCTCF-3 | 5’-GCGCTCTAAGAAAGAAGATTC-3’ |
| shNC | 5’-CAACAAGATGAAGAGCACCAA-3’ |
