# Supplementary material for: Immune alterations and overexpression of CTCF in endometrial carcinoma: insights from molecular subtyping
Source: Cancer Cell Int. 2024 Dec 2;24:392. doi: 10.1186/s12935-024-03576-y (PMC11613940; doi:10.1186/s12935-024-03576-y)
Supplement: Supplementary file 6 — Additional file 6. [file 12935_2024_3576_MOESM6_ESM.docx]

**Table S5. Information on datasets related to the CTCF gene.**

| **Dataset** | **pbgene** | **pb_ENSEMBL** | **nSample** | **celline** | **method** | **accession** |
| --- | --- | --- | --- | --- | --- | --- |
| D20267_CTCF | CTCF | ENSG00000102974 | 4 | Hela | shRNA | GSE108869 |
| D20031_CTCF | CTCF | ENSG00000102974 | 5 | MCF10A | siRNA | GSE101921 |
| D20266_CTCF | CTCF | ENSG00000102974 | 4 | Hela | shRNA | GSE108869 |
| D20571_CTCF | CTCF | ENSG00000102974 | 6 | B-ALL | KO | GSE120781 |
| D22075_CTCF | CTCF | ENSG00000102974 | 6 | Hela | siRNA | GSE79564 |
| D22076_CTCF | CTCF | ENSG00000102974 | 6 | Hela | siRNA | GSE79564 |
| D21087_CTCF | CTCF | ENSG00000102974 | 6 | SLK | siRNA | GSE138937 |
| D21566_CTCF | CTCF | ENSG00000102974 | 8 | HEK293T | siRNA | GSE155541 |
